# Research note: Spatial and temporal distribution of poultry red mite infestations in non-caged barn and free-range laying hen systems

**DOI:** 10.1016/j.psj.2026.106793

**Published:** 2026-03-17

**Authors:** Iram Gladan, Jannigje G. Kers, Mirlin P. Spaninks, Roland G.G. Bronneberg, J.A. Stegeman, Johanna M.J. Rebel, Francisca C. Velkers

**Affiliations:** a Department of Population Health Sciences, Faculty of Veterinary Medicine, Utrecht University, Yalelaan 7, 3584 CL, Utrecht, the Netherlands; b Wageningen Adaptation Physiology Group, Wageningen University and Research, De Elst 1, NL-6708 WD, Wageningen, the Netherlands; c AviVet International B.V., Hoofdstraat 81, 3971 KD, Driebergen-Rijsenburg, the Netherlands

**Keywords:** *Dermanyssus gallinae*, Laying hens, Poultry red mite monitoring, Housing systems, Poultry welfare

## Abstract

Poultry red mite (PRM; *Dermanyssus gallinae)* is a widespread ectoparasite of laying hens, causing major health, welfare, and economic losses. Its nocturnal feeding and concealment in housing structures make infestations difficult to detect and control. PRM control requires insight into spatial and temporal infestation dynamics, which in non-caged laying hen systems may be influenced by housing characteristics, including structural complexity and outdoor access. This study investigated variation in PRM infestations within poultry houses, between housing systems, and over time.

Two aviary-housed laying hen flocks on the same farm, one flock housed indoors and one flock with free-range outdoor access, were monitored longitudinally for 9 months. PRM infestation was assessed every 2 weeks using AviVet® traps placed at multiple locations and quantified by trap weight. In the outdoor house, PRM were first detected at sidewall locations adjacent to the outdoor range and spread throughout the house within 4-6 weeks. After Exzolt® (fluralaner) treatment, the house remained PRM-negative for 3 months. Reinfestation occurred at the same sidewalls, where the highest PRM levels were observed, with trap weights ranging from 0.2 to 473.4 mg per trap. Lower levels (0.1–60.9 mg per trap) were found at the rear, central, and front areas. The indoor house maintained low PRM levels (0.1–16.4 mg per trap).

To examine variation across housing systems, a cross-sectional study was conducted in 6 laying hen houses on 4 farms: 1 indoor house without covered outdoor range, 3 with a covered outdoor range, and 2 organic free-range houses. Three of the 6 houses were PRM-positive (mean trap weights per house: 1922.1, 24.4, and 51.8 mg). PRM distribution varied across flocks, with no significant differences between structural locations.

Overall, PRM infestations in non-caged systems showed substantial spatial and temporal variation within and between houses. These findings highlight the need for structured, multi-location, and repeated PRM monitoring to detect early infestation hotspots and enable timely interventions to support layer health and welfare and reduce economic losses.

## Introduction

Poultry red mite (**PRM**) (*Dermanyssus gallinae*) is the most prevalent ectoparasite in poultry production systems worldwide and is associated with major impacts on flock health, welfare, and economic performance (Sparagano et al., 2009).

Following the European Union’s ban on conventional cages in 2012 (Council Directive 1999/74/EC), egg production systems shifted towards housing systems that allow greater freedom of movement and social interactions among birds. These include enriched cages, indoor (aviary) barn systems, free-range, and organic farming systems (EFSA AHAW Panel, 2023). Although these changes have improved opportunities for natural behaviors, they have also increased the risk of PRM transmission within flocks (Sigognault Flochlay et al., 2017). The rising PRM prevalence in recent years may, therefore, be attributed not only to the growing restrictions on acaricide use, but also to the greater complexity of alternative, non-cage housing systems (Sigognault Flochlay et al., 2017). Barn and free-range aviary systems offer numerous sheltered microhabitats, such as litter, nest boxes, manure belts, and perches, that support PRM survival and reproduction (Decru et al., 2020). In free-range systems, outdoor areas cannot be effectively disinfected, and may, therefore, serve as persistent environmental reservoirs for PRM populations. Due to their nocturnal behavior, PRM hide in cracks and crevices during the day and emerge at night to feed on the hens (Sigognault Flochlay et al., 2017). In multi-tier aviary systems, a manure cross conveyor is located at the rear of the house, where all manure belts from different tiers converge, and manure is transported to an external storage or pit. The location of the manure may provide a sheltered microhabitat conducive to PRM aggregation. Manure-associated structures in non-caged housing systems have previously been reported to harbor large PRM populations (Mul and Koenraadt, 2009). Under optimal environmental conditions, PRM can complete their life cycle within 7 to 10 days, allowing populations to grow rapidly (Nordenfors et al., 1999). Other factors contributing to infestation dynamics include host availability, ventilation, environmental temperature, relative humidity (**RH**), and chemical cues such as ammonia (**NH_3_**) and carbon dioxide (**CO_2_**) (Auffray et al., 2023; Sioutas et al., 2024).

Given the complexity of PRM infestations in modern housing systems, structured, multi-location, and repeated monitoring is crucial for understanding spatial and temporal dynamics of PRM. Sustainable control increasingly relies on Integrated Pest Management (**IPM**), a comprehensive strategy aimed at minimizing chemical use while maximizing long-term pest suppression (Decru et al., 2020). In practice, the two most widely used methods are visual scoring and trap-based monitoring, with the latter shown to provide more sensitive and objective estimates of PRM infestation levels. However, most validation studies have been conducted under experimental conditions or in caged systems with limited evidence from structurally complex barn or free-range systems (Lammers et al., 2017; Oh et al., 2020).

This study aimed to assess the spatial and temporal distribution of PRM in barn and free-range laying hen housing systems under commercial field conditions, taking potential effects of structural characteristics and house climate into account. Spatial distribution was evaluated longitudinally in 2 aviary houses on a single farm (one indoor and one with outdoor free-range access) and cross-sectionally in 6 laying hen houses on 4 farms across different housing systems, including barn, free-range, and organic systems. Specifically, we differentiated between the rear of the house, near the manure cross conveyor (rear end), the left and right lateral sidewalls of the house (sidewall locations), and other locations (central or front sections of the house).

## Materials and methods

### Ethics statement, farm selection and consent

This field study involved non-invasive sampling and observational data collection conducted during routine farm management and standard veterinary procedures. No experimental treatments or interventions were applied. Following consultation with the institutional animal welfare body, the study was confirmed not to constitute animal experimentation under Dutch legislation (EU Directive 2010/63/EU). Farms were selected using convenience sampling, based on the farmers’ willingness and interest to participate in a study on PRM. Farmers were informed about the study objective and provided written informed consent prior to data collection. All data were anonymized and handled in accordance with the General Data Protection Regulation.

### Farm and flock characteristics

Two houses on one of the farms (A-I, an indoor house; and A-II, a house with outdoor free-range access) were studied longitudinally at two-week intervals between 22 March 2024 and 8 November 2024. Both houses had a similar internal layout and functional design, water was supplied via nipple drinkers, and feed distribution was automated and synchronized between houses. The stocking density was 9 hens/m^2^, with in A-II an additional 4 m^2^/hen free-range area. Six additional houses on 4 farms (B, C, D, and E) were visited for sampling and data collection at a single time point (cross-sectional study) in the study period. All 8 houses across 5 farms were equipped with open multi-tier aviary systems including integrated nest boxes, perches, and manure belts converging at a rear cross conveyor. Six houses (A-II, C-I, D-I, E-I, E-II, and E-III) included a covered outdoor range (“wintergarten”), and 3 (A-II, C-I, D-I) provided additional access to an uncovered free-range area, of which 2 (C-I and D-I) operated under organic management. Table 1 summarizes the main housing and flock characteristics of all flocks, houses, and farms.

**Table 1:** Overview of housing types, flock characteristics, PRM infestation status, and timing and type of PRM control methods in the longitudinal (2 houses farm A) and cross-sectional (6 houses, 4 farms) studies.

Cross-sectional study							Longitudinal study	
House^1^	B-I	C-I	D-I	E-I	E-II	E-III	A-I	A-II
Flock specifics^2^								
Housing system	Barn	Free-range	Free-range	Barn	Barn	Barn	Barn	Free-range
Housing type	Indoor	Outdoor	Outdoor	Indoor plus	Indoor plus	Indoor plus	Indoor	Outdoor
Uncovered outdoor range	No	Yes^++^	Yes^+^	No	No	No	No	Yes^+^
Covered outdoor range (wintergarten)	No	Yes^++^	Yes^+^	Yes^+^	Yes^+^	Yes^+^	No	Yes^++^
Flock data								
Hen breed^3^	LSL	DW	LB	LSL	LSL	LSL	DW	DW
Number of hens^4^	39,094	17,863	19,000	22,277	24,541	23,943	13,295	26,822
Bird density (hens/m²)	9	6	6	9	9	9	9	9^5^
Age (weeks)	74	49	50	77	93	93	22-55	40-69
Poultry red mite								
PRM infestation^6^	Yes	No	Yes	No	No	Yes	Yes	Yes
PRM control^7^	Silica	Q-perch	Q-perch	Exzolt®^9^	None^8^	None^8^	Exzolt®^9^	Exzolt®^9^
Number of traps placed	20	10	10	10	10	10	10	20
Months since last PRM control	2.5	Continuous	Continuous	3	NA	NA	NA	NA

1 Letters (A, B, C, D, E) indicate the farm; Roman numerals (I, II, III) distinguish individual houses within the farm.

2 All houses used an open, multi-tier aviary system. Outdoor = free-range access. Indoor plus = with covered outdoor range (i.e. a ’wintergarten’). Indoor = without wintergarten and without free-range. Wintergarten or outdoor access on one or both sidewalls is indicated by + or ++. The two free-range houses (C-I and D-I) also used an organic production system.

3 LSL = Lohmann Selected Leghorn; DW = Dekalb White; LB = Lohmann Brown.

4 Number of hens present at the first sampling point.

5 The free-range area for house A-II provided space for 4 hens per m^2^.

6 PRM detection using AviVet® PRM traps to measure PRM weight (mg) and additional Mite Monitoring Score (MMS) for visual assessment of infestation levels.

7 PRM control measures applied.

8 No PRM treatment during this production cycle.

9 Exzolt® (fluralaner) was used as treatment against PRM infestations.

Of the 8 houses, 5 showed signs of PRM infestation (A-I, A-II, B-I, D-I, and E-III). Different PRM control strategies were used across farms. Fluralaner treatments (Exzolt®, Intervet International B.V., Boxmeer, the Netherlands) were administered in A-I at week 34 and for A-II at week 46 of age via the drinking water. In house D-I, Exzolt® was administered via drinking water 3 months prior to our sampling visit. In house B-I, silica (a desiccant powder that physically damages and dehydrates PRMs) was applied 2.5 months before sampling. A Q-perch® system (Vencomatic, Eersel, the Netherlands), which features low electric current through perch surfaces, was used as a continuous PRM control method in houses C-I and D-I. No PRM control measures had been applied in E-II and E-III at the time of sampling (Table 1).

### Flock monitoring and measurements

**Fig. 1:**
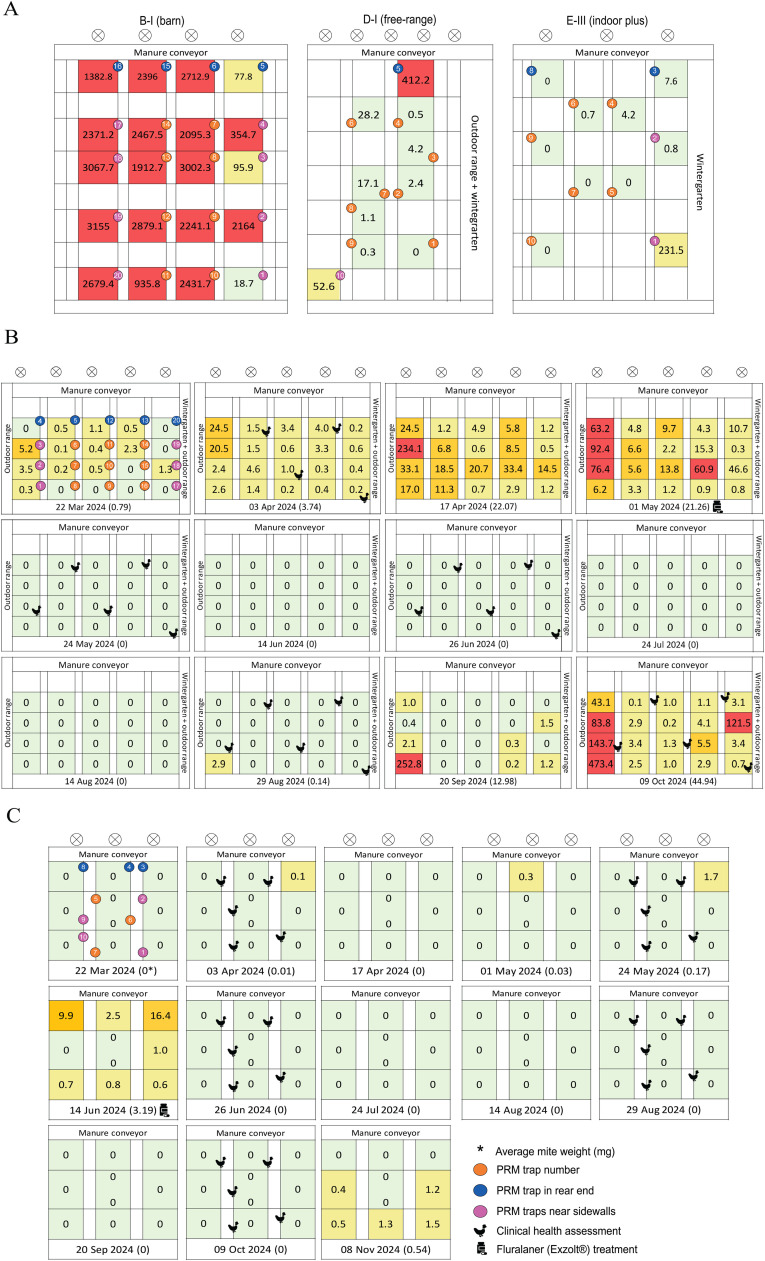
Overview of the spatial and temporal progression of poultry red mite (PRM) infestation in (A) cross-sectional PRM-positive houses (B-I, D-I, E-III), (B) longitudinal outdoor house (A-II), and (C) longitudinal indoor house (A-I). (A) Heatmaps show AviVet® trap weights (mg) at individual sampling compartments within PRM-positive houses included in the cross-sectional study. Each colored cell represents a sampling compartment where a PRM trap was placed, with numbers indicating trap weights (mg). Infestation levels were classified according to AviVet® thresholds as low (green: ≤50 mg), medium (orange: 51–250 mg), and high (red: >250 mg). (B–C) Heatmaps show AviVet® trap weights (mg) across repeated sampling time points in the longitudinal outdoor house (A-II) and longitudinal indoor house (A-I). A custom color scale was used to visualize temporal changes in infestation intensity: 0–5 mg (light yellow), 5–50 mg (orange), and >50 mg (red). Sampling points are indicated along the central timeline, with sampling date and average PRM trap weight (mg) shown in brackets (*). For A-II, the first sampling occurred at 40 weeks of age; for A-I, at 22 weeks of age. Medicine icons indicate Exzolt® treatment. In A-II, treatment was administered on 4 May 2024 (hens 46 weeks of age), three days after sampling at time point 4. In A-I, treatment was administered on 15 June 2024 (hens 34 weeks of age), one day after sampling at time point 6. Trap numbers are color-coded by location (orange: front and middle compartments; blue: rear end; purple: sidewalls). Chicken icons indicate locations where health, welfare, and behavioral assessments were performed. Farm structural features, including ventilators, wintergarten, and the central manure cross conveyor, are marked on each heatmap for spatial reference.

To study the potential influence of proximity to structural locations on the spatial distribution of PRM quantities, trap positions were identified and mapped in schematic blueprints for the longitudinal and PRM-positive cross-sectional houses (Fig. 1). The PRM-negative houses, i.e. C-I (outdoor with wintergarten and free-range), E-I and E-II (both indoor plus), had comparable structural layouts and housing designs to other houses of the same system. Locations were categorized as rear end (rear of the house, near the manure cross conveyor), sidewalls (left and right lateral sidewalls), and other locations (central or front sections of the house). In farm A, internal fences divided houses into compartments, limiting movement between compartments while allowing horizontal and vertical movement within each multi-tier aviary. In the free-range house (A-II), pop-holes provided daytime access to the outdoor range.

PRM infestations were quantified based on the weights of the PRM contents collected in AviVet® PRM traps (Lammers et al., 2017). This experimentally validated method has been widely used for routine monitoring of PRM in laying hen houses, including in an EU monitoring research project (Interreg NWE, 2020), and is considered a suitable method for routine monitoring of PRM on farms. Traps were evenly distributed and positioned near hens’ resting areas following established monitoring protocols (Decru et al., 2020). Depending on flock size 10 traps (≤25,000 hens) or 20 traps (>25,000 hens) were placed per house to ensure proportional spatial coverage. After 48 hours, traps were retrieved, individually sealed in plastic bags, stored at −20 °C and weighed to determine total PRM load across all life stages (nymphs, adults, larvae, and eggs).

Visual scoring was performed using the Mite Monitoring Score (**MMS**; 0–4 scale) as a complementary qualitative assessment alongside trap quantification (Interreg NWE, 2020). MMS primarily reflects infestation trends but is less sensitive to low mite populations (Mul et al., 2015; Oh et al., 2020). MMS rates infestation severity on a 0–4 scale per m²: 0 = no mites visible; 1 = mites in cracks or crevices; 2 = mites at exposed sites; 3 = clusters (> 1 cm²) in cracks or crevices; 4 = clusters (> 1 cm²) at exposed sites. PRM assessments were conducted once in the cross-sectional study and every two weeks in the longitudinal study (13 time points in A-I and 12 in A-II).

Additional measurements included climate and flock health monitoring. On farm A, temperature, RH, CO₂, and NH₃ were recorded every 10 min throughout the 9-month monitoring period using the Intelligent Barn System® (Connecting Agri and Food B.V., Uden, the Netherlands). In the cross-sectional houses, climate parameters were measured once at each PRM trap location using a Testo 300 flue gas analyzer (Testo SE and Co. KGaA, Germany).

Production parameters were obtained from flock management software, and flock health and welfare were assessed periodically on farm A using a structured laying hen assessment protocol (KipUP; Fair Poultry, NL), including visual scoring of comb color and feather condition, and additional individual scoring of skin lesions, comb damage, and body weight (see Supplementary Methods in Supplementary Material S1). On cross-sectional farms, production data and general observations of health, behavior, and welfare were recorded once during trap placement.

### Data handling, analysis and statistics

Raw data were organized and preprocessed in Microsoft Excel and analyzed in R (version 4.2.3), using the packages ggplot2, ggpubr, dplyr, tidyr, and readxl. Spatial heatmaps and timelines were created with an online graphic design tool Canva (Canva Pty Ltd., Sydney, Australia) and finalized using Adobe Illustrator version 29.8.2 (64-bit) (Adobe Inc., USA).

In the cross-sectional study, infestation levels were categorized according to AviVet® trap guidelines (≤50 mg low, 51–250 mg medium, >250 mg high) (Lammers et al., 2017) and visualized using a green/light yellow–orange–red gradient, scaled per farm to reflect within-house variation in infested houses. In the longitudinal study, where infestation levels were generally low, customized gradients (0–5 mg light yellow, 5–50 mg orange, >50 mg red) were applied to capture subtle spatial and temporal variation.

To evaluate differences in PRM levels between structural locations, traps were grouped into 3 categories based on their placement within each house: rear (near the manure cross conveyor), sidewalls (left and right), and other (central or front sections). For the longitudinal houses (A-I, A-II), only pre-treatment measurements were included, while single time-point measurements were used for the infested cross-sectional houses (B-I, D-I, E-III). To assess whether PRM infestation was consistent across structural locations within houses, a Friedman rank-sum test was performed with house as the blocking factor, using one median PRM value per structural location per house (3 values per house). All tests were two-sided, and statistical significance was set at *P* < 0.05. For visualization purposes, PRM weights in the cross-sectional study were displayed on a log₁₀ (PRM weight + 1) scale, to accommodate the presence of zero values.

## Results and discussion

This study examined the spatial and temporal dynamics of PRM across indoor and outdoor commercial laying hen houses, considering the potential influence of structural features such as the rear end near the manure cross conveyor, lateral sidewalls with/without outdoor access, and central/front sections on PRM levels.

In the longitudinal study, contrasting patterns were observed between the indoor and outdoor houses. The indoor house (A-I) remained largely PRM-free (Fig. 1C). Only intermittent low-level detections were observed (0.1–16.4 mg), remaining well below established low infestation thresholds (≤ 50 mg) (AviVet®; Interreg NWE, 2020).

In contrast, the outdoor house (A-II) showed recurring infestations of varying intensity (Fig. 1B). During the initial sampling period (weeks 40–44), infestations were consistently concentrated along the left sidewall adjacent to the outdoor range. By week 46, all traps were positive, with the highest loads recorded near the outdoor access point.

Following Exzolt® treatment (after time point 6 in A-I and time point 4 in A-II), PRM levels remained undetectable for approximately three months in both longitudinal houses (Supplementary Figs. S2A, S2B). However, post-treatment dynamics differed spatially and temporally between houses. In the outdoor house (A-II), PRM infestation re-emerged at time point 10 (17 weeks post-treatment), first at sidewall locations adjacent to the wintergarten/outdoor access (Supplementary Fig. S2B), suggesting that these areas may act as localized refugia or potential reinfestation routes associated with outdoor access, as described previously (Mul and Koenraadt, 2009). By time point 11, 20 weeks post-treatment, increases were also observed at the rear end, while other locations showed only minor changes. Infestation levels continued to rise thereafter. At the final sampling time point, all traps were positive, with the highest PRM loads consistently recorded along the sidewall adjacent to the outdoor range, and values exceeding the high infestation levels of 250 mg threshold only at this location.

In contrast, the indoor house (A-I) remained largely PRM-free throughout the post-treatment period. Only minimal fluctuations were observed at sidewall locations from time point 13 (21 weeks post-treatment) onwards, with no resurgence at the rear end or other areas (Supplementary Fig. S2A). The post-treatment dynamics differed clearly between A-I and A-II, indicating that PRM persistence and re-emergence were house-specific rather than consistently linked to specific structural areas. The generally low infestation levels, combined with early fluralaner treatment, likely suppressed PRM population development during the warmest period, when growth is typically favored (Nordenfors et al., 1999). This may have limited the ability to detect location-specific aggregation.

Three of the 6 houses in the cross-sectional study were PRM-positive (B-I, D-I, and E-III), while C-I, E-I, and E-II remained PRM-negative. Heatmaps revealed spatial heterogeneity both within and between housing systems (Fig. 1A), consistent with previous observations of uneven spatial distribution of PRM within poultry houses (Sioutas et al., 2024).

In the indoor house (B-I), infestation was high and widely distributed across locations. The highest trap weights were observed in central areas and along the left sidewall. In the free-range house (D-I), infestation levels were generally low, with a single high value recorded at the rear end near the manure cross conveyor (412.2 mg). In E-III (indoor with wintergarten), infestation levels were also low overall, except for one elevated trap weight at the wintergarten along the right sidewall (231.5 mg), while rear-end locations remained PRM-negative or showed low positive values (Supplementary Fig. S3).

The spatial patterns observed in trap weights were not reflected by visual scoring. Locations with high PRM weights did not consistently correspond to high visual scores, indicating limited agreement between the two methods (Supplementary Table S1). This discrepancy is consistent with previous research showing that visual assessment underestimates PRM presence, particularly at low infestation levels or in less accessible areas (Oh et al., 2020). A review similarly concluded that, although practical, visual scoring lacks sensitivity for early detection (Decru et al., 2020). In line with the generally low PRM infestation levels, the hens on the farm in the longitudinal study remained in good clinical condition. Production performance remained within expected breed standards, and health and welfare parameters, including comb color and condition and overall KipUP scores, showed only limited (age-associated) feather damage unrelated to PRM levels (Fig. S1A–B in Supplementary Material S1). In the cross-sectional study, flocks also performed within expected ranges and showed normal behavior and no major health concerns, except for isolated pale combs and feather loss in one house (B-I).

Environmental conditions, particularly temperature and relative humidity, have been reported to influence PRM development, survival, and activity (Nordenfors et al., 1999; Auffray et al., 2023). In the longitudinal houses, climate parameters generally remained within recommended industry ranges for laying hens (EFSA AHAW Panel, 2023), with occasional exceedances of temperature, CO₂, or NH₃ thresholds (Supplementary Fig. S4). In the cross-sectional houses, single measurements at trap locations showed moderate spatial variation but also remained largely within standard industry ranges. No consistent association was observed between climatic parameters and PRM infestation levels (Supplementary Fig. S5; Table S2).

All houses consisted of comparable multi-tier aviary systems, and differences in PRM levels between farms were not associated with housing system type. This finding aligns with a European prevalence study reporting similar infestation levels across cage and barn systems (Sparagano et al., 2009), suggesting that farm and flock management, biosecurity, and control measures may influence PRM dynamics more than housing type alone. Moreover, no structural location showed consistently higher PRM levels across houses in our study, and statistical analyses did not reveal significant differences between rear, sidewall, and other locations. Previous studies, however, have suggested that PRM infestations are more common in barn and free-range houses than in enriched cages, likely due to increased structural complexity and availability of hiding places (Sparagano et al., 2009). Structural differences, such as multi-tiered aviaries versus single-level cages, have also been associated with variation in infestation levels (Mul and Koenraadt, 2009). Together, these findings indicate that the progression of PRM infestations is difficult to predict. Consistent with Integrated Pest Management principles (Decru et al., 2020), continued field-based validation of monitoring protocols under commercial conditions remains essential. Effective PRM control requires systematic monitoring and post-treatment evaluation, particularly in areas prone to recurring persistence. Emerging technologies, including sensor-based and AI-driven monitoring tools, may enhance continuous detection and support earlier, more targeted control strategies. Strengthening PRM monitoring remains critical for timely intervention and protection of bird health and welfare.

## Funding

This project has received funding from the research program Nationale Wetenschapsagenda – Onderzoek op Routes door Consortia (NWA-ORC) 2020/21 which is (partly) financed by the Dutch Research Council (NWO) under number NWA.1389.20.123.

## Acknowledgements

The authors of this paper would like to express their appreciation for the efforts applied in data collection by the management and staff of the layer farms, as well as Lex Kneppers and Peter van de Laar from van Eck Bedrijfshygiëne B.V. (Son, the Netherlands) for their cooperation with this research.

## Data availability

The datasets and R scripts used for data analysis and figure generation in this study, together with the accompanying Excel files required to reproduce the analyses, are available in the Zenodo repository at https://doi.org/10.5281/zenodo.18242218.

## Declaration of generative AI and AI-assisted technologies in the manuscript preparation process

During the preparation of this work, the authors used ChatGPT (OpenAI, GPT-5.2) to assist with language editing and grammar refinement. After using this tool/service, the authors reviewed and edited the content as needed and take full responsibility for the content of the published article.

## CRediT authorship contribution statement

**Iram Gladan:** Writing – review & editing, Writing – original draft, Visualization, Validation, Methodology, Investigation, Formal analysis, Data curation, Conceptualization. **Jannigje G. Kers:** Writing – review & editing, Visualization, Validation, Supervision, Software, Methodology, Formal analysis, Data curation, Conceptualization. **Mirlin P. Spaninks:** Writing – review & editing, Validation, Resources, Investigation, Data curation. **Roland G.G. Bronneberg:** Writing – review & editing, Validation, Resources, Methodology, Investigation, Conceptualization. **J.A. Stegeman:** Writing – review & editing, Supervision, Funding acquisition, Conceptualization. **Johanna M.J. Rebel:** Writing – review & editing, Supervision, Funding acquisition, Conceptualization. **Francisca C. Velkers:** Writing – review & editing, Visualization, Validation, Supervision, Resources, Project administration, Methodology, Investigation, Funding acquisition, Formal analysis, Data curation, Conceptualization.

## Disclosures

The authors of this manuscript declare that they have no known competing financial or non-financial interests that could have influenced the work reported in this manuscript.

## Appendix Supplementary materials

Image, application 1

Image, application 2

Image, application 3

Image, application 4

Image, application 5

Image, application 6

Image, application 7

Image, application 8

## Footnotes

Supplementary material associated with this article can be found, in the online version, at doi:10.1016/j.psj.2026.106793.
