# Supplementary material for: Research note: Spatial and temporal distribution of poultry red mite infestations in non-caged barn and free-range laying hen systems
Source: Poult Sci. 2026 Mar 17;105(6):106793. doi: 10.1016/j.psj.2026.106793 (PMC13068535; doi:10.1016/j.psj.2026.106793)
Supplement: Supplementary file 1 [file mmc1.pdf]

Supplementary Methods

For Research Note: Spatial and temporal distribution of poultry red mite infestations in non-caged barn and free-range laying hen systems, Iram Gladan et al.

Flock performance and clinical health and welfare indicators

In the longitudinal study, the diagnostic procedures and PRM control measures were part of standard veterinary procedures in accordance with national veterinary regulations and standards for food safety applicable to laying hens. This was performed under the guidance of experienced and certified poultry veterinarians. These included treatments with Exzolt® (Intervet International B.V., Boxmeer, the Netherlands) with the active substance fluralaner. Exzolt® was administered in the flock in A-I at week 34 and for A-II in week 46 via the drinking water for PRM control. For both flocks of farm A, production parameters (egg production, mortality, feed and water intake, and average egg weight) were obtained from a digital flock management software program (PoultryPlan, Ede, the Netherlands). Hen-housed egg production (HHEP) and hen-day egg production (HDEP) were benchmarked against the Dekalb White free-range standards (Hendrix Genetics, Boxmeer, the Netherlands). Production data of the barn (A-I) were available until week 53, while PRM assessment continued until week 55. For the flock health assessment, flock health, behavior, welfare, and environmental conditions were evaluated using a structured laying hen assessment protocol (KipUP; Fair Poultry, the Netherlands). This assessment was conducted in the barn (A-I) and free-range (A-II) house at five time points, with an average interval of 6.5 weeks between visits, with the first assessment in week 24 (A-I) and week 42 (A-II), and the last in week 51 (A-I) and week 69 (A-II). Individual-based health and welfare parameters of the KipUP protocol relevant to PRM infestations were scored. Comb color and feather condition were visually scored in 100 hens without handling (10 hens at each of the 10 locations, where also PRM traps were placed). The individual scores were recorded with reference to the PRM trap number to allow for combined analyses. Additionally, 50 other hens were caught from different locations of the same house to assess health parameters that required handling for inspection. These included, among others, skin lesions and comb damage. The individual body weight of these hens was recorded using a digital scale (Kern HDB 5K5N, Kern & Sohn GmbH, Balingen, Germany; precision 0.005 kg). Environmental conditions were also evaluated with KipUP, and additionally with parts of the Welfare Quality protocol for laying hens, i.e., assessing thermal comfort, water access, and litter quality (Welfare Quality®, Lelystad, the Netherlands). Temporal changes in feather condition and comb color, alongside average PRM trap weight and flock mortality, are presented in Supplementary Figures S1A and S1B.

Data analysis

To determine whether changes in PRM infestation coincided with changes in health or welfare parameters, 5 data sources (trap weights, feather and comb color scores from visual assessments at PRM trap locations; clinical parameters from the handled hens; and production records) were standardized to ISO weeks and aggregated at the weekly level.

The individual clinical parameters that required handling, including skin lesions and comb damage, were excluded from further analyses due to their low occurrence, lower sampling coverage compared with visual assessments, and the absence of an association with overall PRM infestation at house level based on descriptive statistics and data visualization. In addition, flock-level behavioral and environmental parameters from the KipUP protocol did not show any relation with the PRM levels in the houses throughout the study and were therefore not analyzed further.

Results

In indoor flock A-I (22–55 weeks of age), PRM infestation levels remained consistently low, and hens remained in good clinical condition. Egg production was according to performance standards. Body weights at all 5 time points of clinical scoring were within the performance standard range (1.50–1.70 kg per hen) (Hendrix Genetics, 2020). Weekly mortality remained low, with cumulative mortality reaching 1.5% by week 53. Limited feather damage and comb color changes were recorded with the KipUP protocol (Supplementary Figure S1A).

Hens in outdoor house A-II (40–69 weeks of age) showed a higher and more variable PRM infestation compared to A-I and experienced some health problems during the study. These did not affect egg production, which remained according to the production standards. Average body weights were stable and appeared even slightly above the Dekalb White performance standards across all measured time points, i.e. 1,700 g in week 43 and 69 (Hendrix Genetics, 2020). In contrast, mortality spiked above performance standards from week 49 onwards, due to *Escherichia coli* (*E. coli*) sepsis, confirmed by bacteriological examination. Subsequently, this was treated successfully with colistin sulfate (Colistin®, Dopharma, Raamsdonksveer, the Netherlands), and in week 54, a live *E. coli* vaccine (Poulvac®, Zoetis, Capelle aan den IJssel, the Netherlands) was applied to provide future immunity. Peaks in mortality were clearly linked to the *E. coli* infection and not related to PRM infestation dynamics, and no association was found between PRM levels and pale combs or feather damage, or other indicators of impaired health or welfare (Supplementary Figure S1B). Parameters evaluated during individual handling, such as comb damage, likewise showed no relationship with PRM infestation and are not shown. Thermal comfort, water availability, and litter quality assessments (adapted from the Laying Hen Welfare Protocol, Welfare Quality®, 2009) indicated satisfactory conditions in both houses across the study.

References

Welfare Quality®, 2009. Welfare Quality® Assessment Protocol for Poultry (broilers, laying hens). Welfare Quality® Consortium, Lelystad, Netherlands. Accessed Oct. 2025: https://www.welfarequalitynetwork.net/media/1293/poultry-protocol-watermark-6-2-2020.pdf

PoultryPlan. 2025. PoultryPlan flock management software. PoultryPlan, Ede, The Netherlands. Accessed Oct. 2025. https://poultryplan.com

Hendrix Genetics. 2020. Dekalb White free-range performance standards. Hendrix Genetics, Boxmeer, The Netherlands. Accessed Oct. 2025. https://www.dekalb-poultry.com/en/product/dekalb-white/

Figure S1A. Feather and comb color changes in relation to average poultry red mite (PRM) weight of the indoor house (A-I) over time.

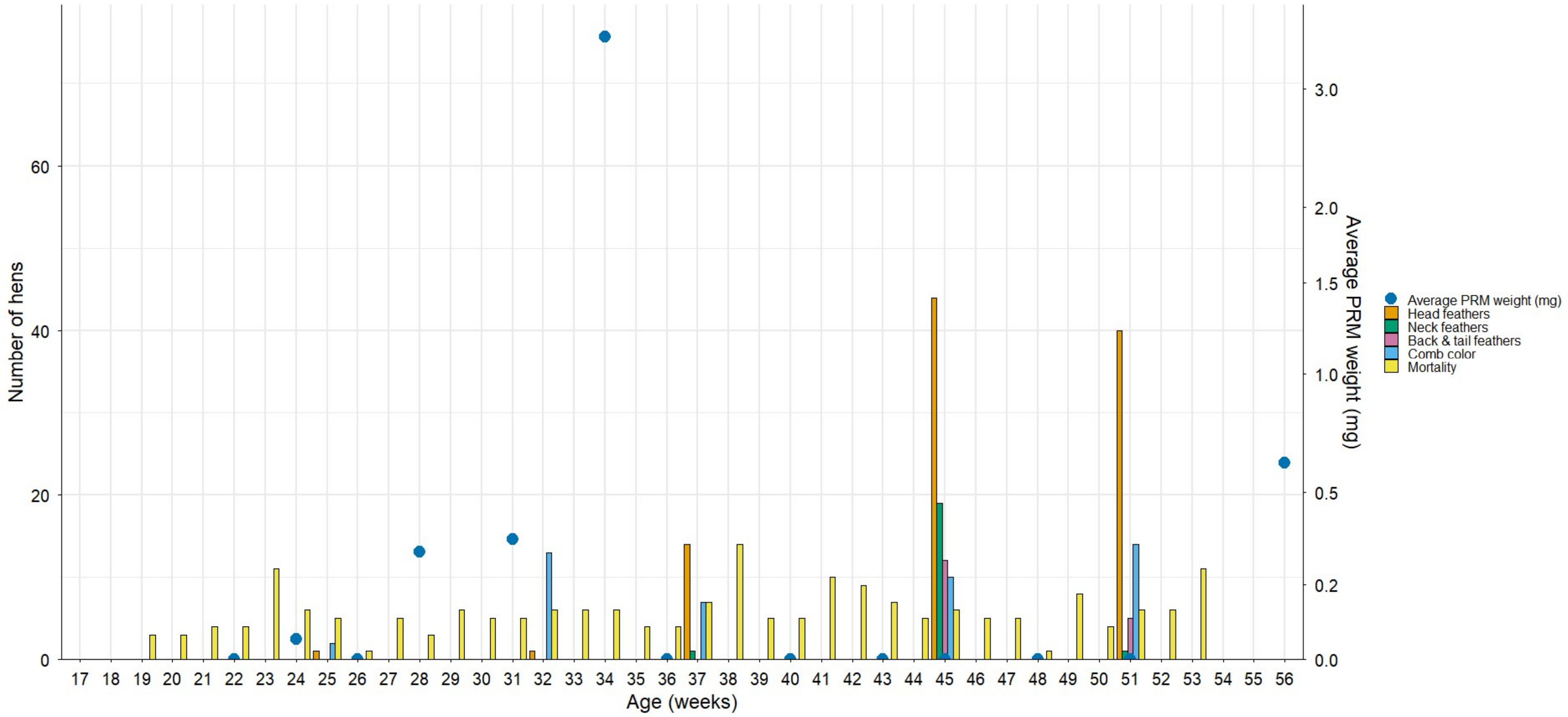

Feather and comb condition were assessed during health, behavior and welfare assessments (n = 100 hens per assessment; 10 hens at 10 locations; 5 assessments in total). Bars represent the number of hens showing abnormalities. Mortality data were obtained from routine production records.

Figure S1B. Feather and comb color changes in relation to average poultry red mite (PRM) weight of outdoor house (A-II) over time.

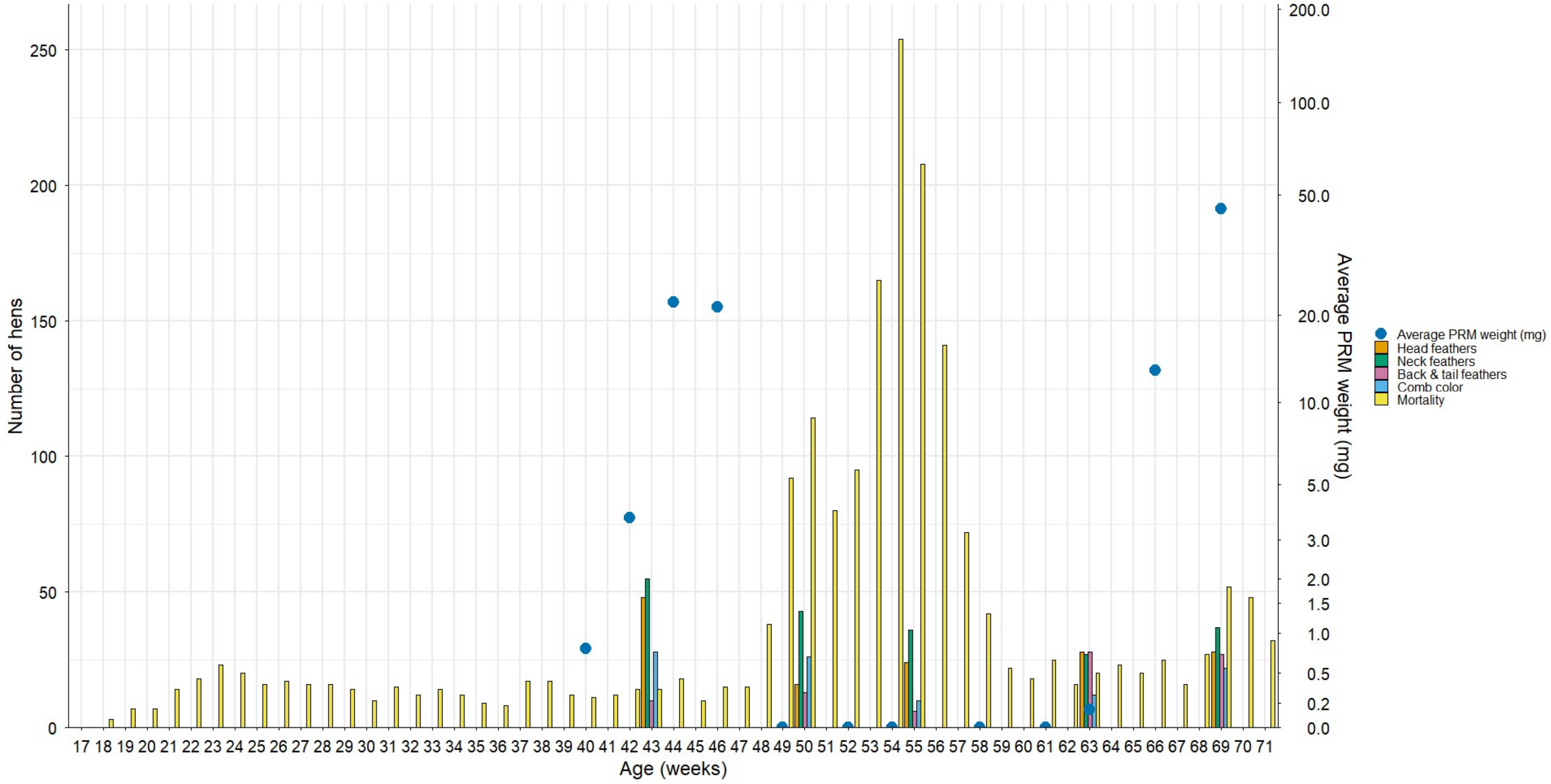

Feather and comb condition were assessed during health, behavior and welfare assessments (n = 100 hens per assessment; 10 hens at 10 locations; 5 assessments in total). Bars represent the number of hens showing abnormalities. Mortality data were obtained from routine production records
