# Supplementary figures and images for: Research note: Spatial and temporal distribution of poultry red mite infestations in non-caged barn and free-range laying hen systems
Source: Poult Sci. 2026 Mar 17;105(6):106793. doi: 10.1016/j.psj.2026.106793 (PMC13068535; doi:10.1016/j.psj.2026.106793)

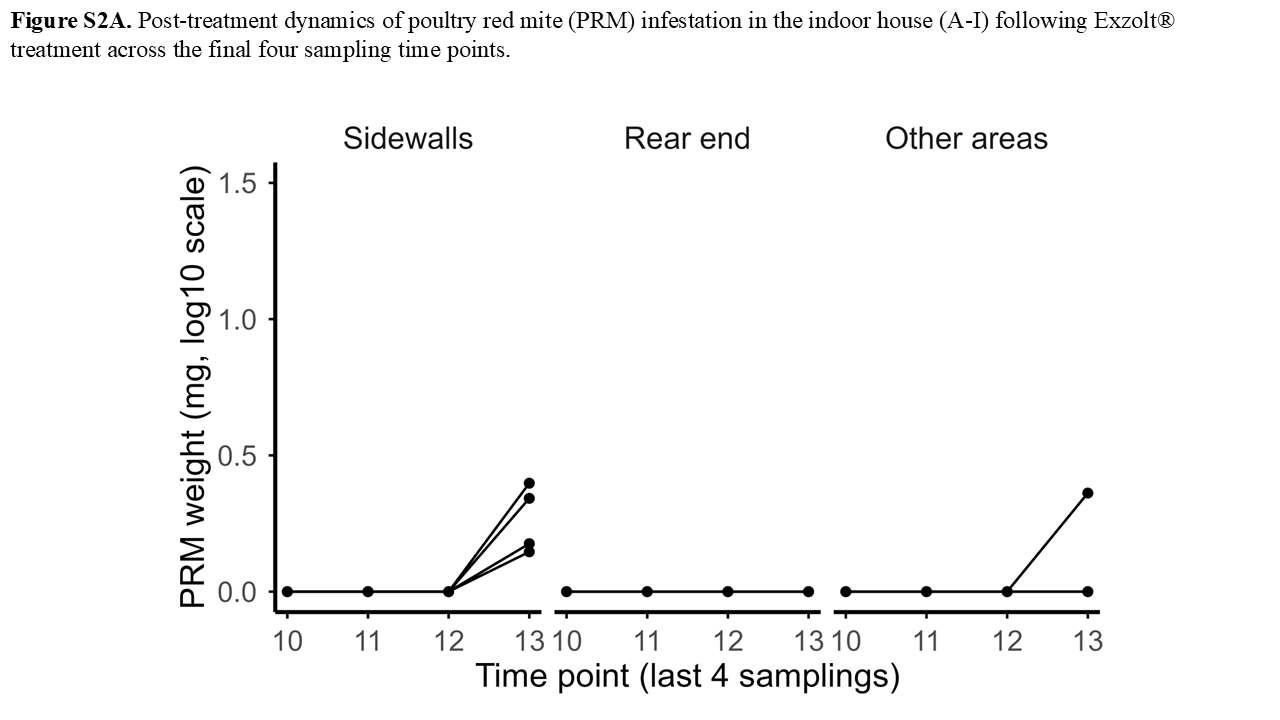

Supplement: Supplementary file 2 [file mmc2.docx]

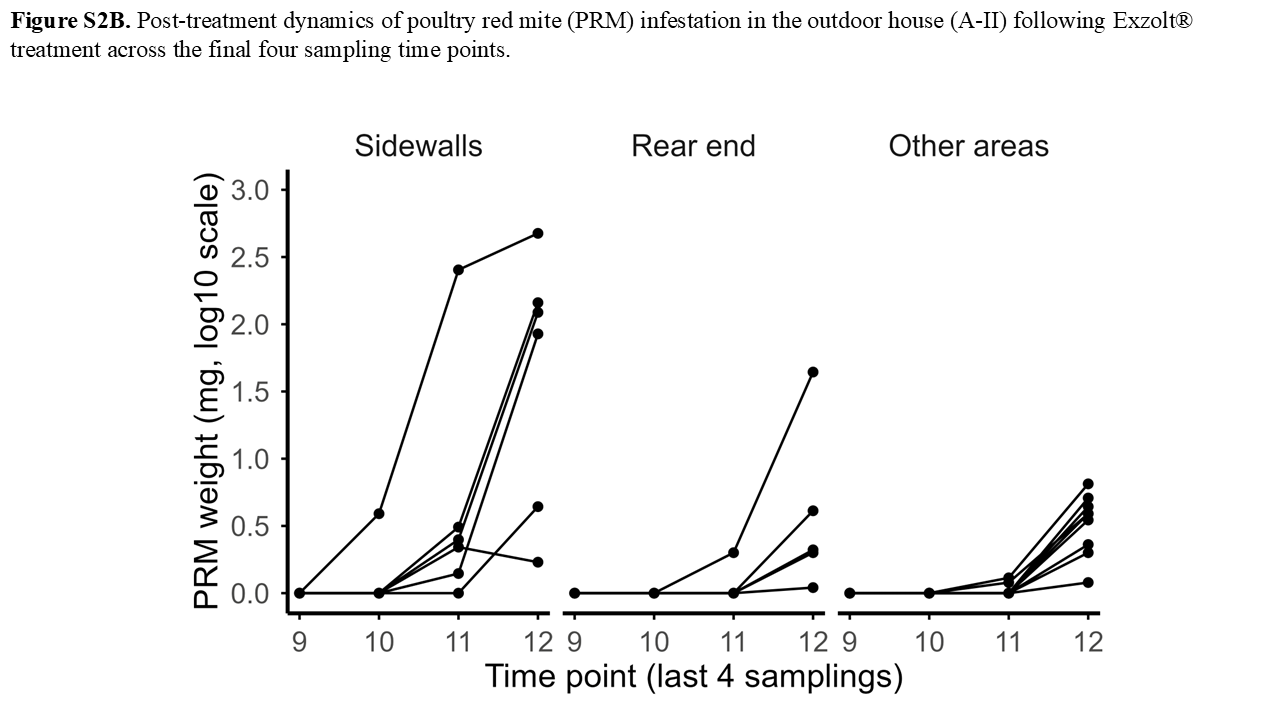

Supplement: Supplementary file 3 [file mmc3.docx]

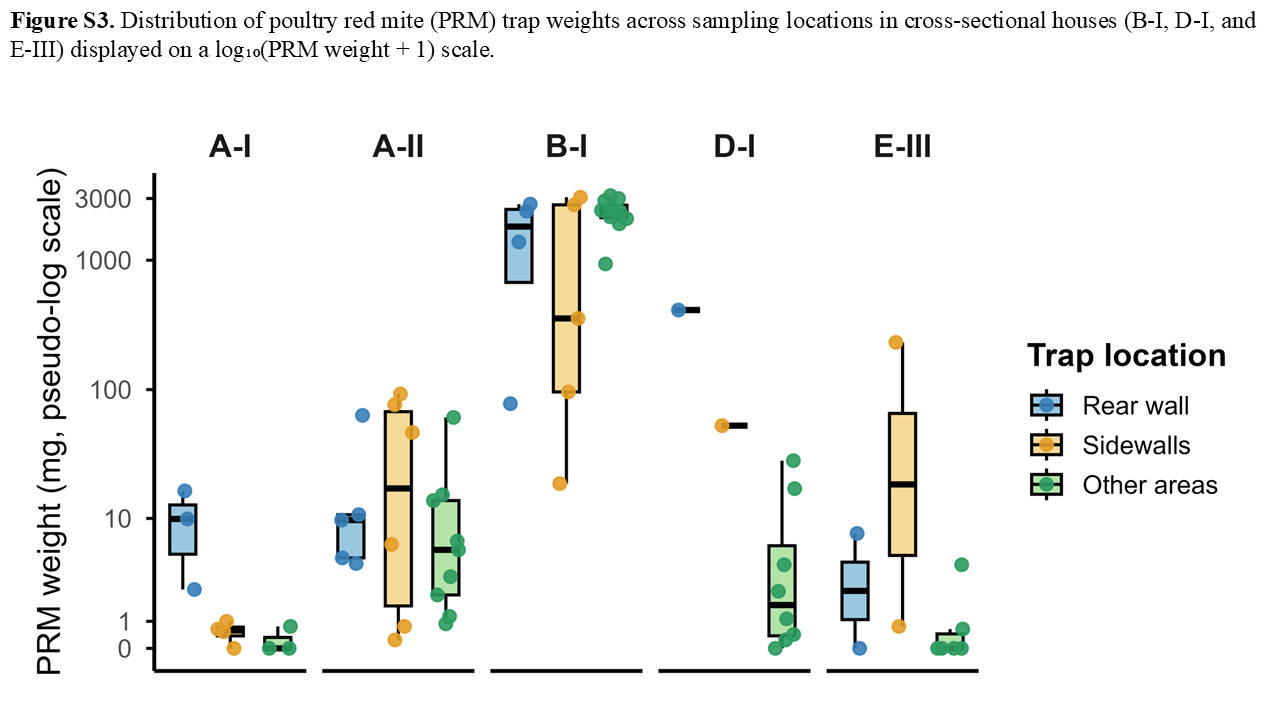

Supplement: Supplementary file 4 [file mmc4.docx]

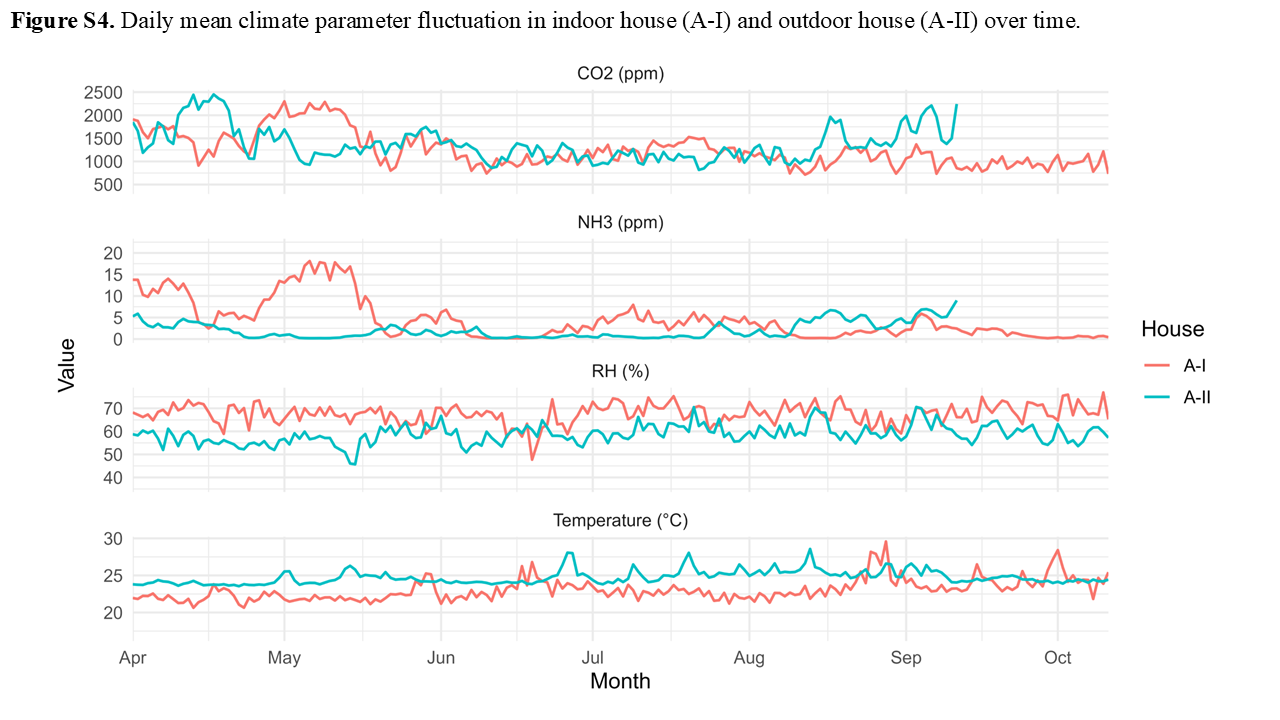

Supplement: Supplementary file 5 [file mmc5.docx]
